# The Relationship Between Sleep Quality and Quality of Life Among Patients With Asthma

**DOI:** 10.7759/cureus.23402

**Published:** 2022-03-22

**Authors:** Farhad Malek, Shokufe Khalil Sayah, Naim Sadat Kia, Elahe Ghods

**Affiliations:** 1 Internal Medicine, Kosar Hospital, Semnan University of Medical Sciences, Semnan, IRN; 2 Community Medicine, Research Center for Social Determinants of Health, Faculty of Medicine, Semnan University of Medical Sciences, Semnan, IRN; 3 Community Medicine, Research Center for Social Determinants of Health, Semnan University of Medical Sciences, Semnan, IRN

**Keywords:** pittsburgh sleep quality index, asthma, sleep quality, sf 36, quality of life

## Abstract

Background and objective

Asthma is a chronic recurring respiratory disease, and its prevalence is on the rise. A drop in the quality of life (QoL), as well as sleep problems, has been reported in asthmatic patients in the literature. This study was conducted to determine the relationship between QoL and sleep quality in asthmatic patients.

Methods

This cross-sectional study recruited 76 diagnosed asthmatic outpatients from our university clinic. The subjects completed the self-administered 36-Item Short-Form Health Survey QoL questionnaire (SF-36) and the Pittsburgh Sleep Quality Index (PSQI) assessment.

Results

The study results revealed poor sleep quality in 55 (72.4%) of the subjects. The total sleep quality scores significantly and negatively correlated with the physical and mental components of QoL (moderate, p=0.00). All QoL subscales were significantly higher in patients with good sleep quality (PSQI: <5, p<0.00). Logistic regression analysis showed that good sleep quality (modified) could predict a mental component score (MCS) of more than 64.

Conclusion

Poor sleep quality can bring down the scores of all QoL components in asthma patients and is the best predictor of their QoL. Therefore, sleep quality and QoL indicators could be applied as part of a better approach to classification, management, and control of asthma.

## Introduction

Asthma is one of the most common chronic diseases, and it causes a noticeable burden on healthcare systems worldwide [1-4], as well as in Iran [5]. Asthma diagnoses have increased in the past three decades due to changes in lifestyle and increasing environmental pollution [1,2,5]. Asthma affects not only the physical but also the psychological aspects of patients' lives [6]. Therefore, the management of symptoms and control of pathologic changes of the disease in clinical settings is not adequate [7]. Qualitative health indicators and asthmatic patients’ perception of their condition - quality of life (QoL) - have been well-addressed over the past three decades [6,8-10].

Asthma patients experience several limitations in their daily activities, productivity, physical and emotional capacity [1,2,6,11], and especially sleep quality [4,12,13]. QoL in asthmatic patients is affected by several factors, such as gender, socioeconomic status, education level, and place of residence [3,9,14]. Numerous studies have shown that QoL indices have significant determining effects on the classification, diagnosis, and treatment of asthmatic patients [6,15]. Although there are various tools for the assessment of QoL [8,10,15,16], the 36-Item Short-Form Health Survey QoL questionnaire (SF-36) is one of the most commonly used tools in this field [15,17-19]. It is well-established and appropriate for evaluating the effects of chronic diseases and their treatments on QoL [7,10,20,21] and hence could have some role in measuring the burden of the disease [3,9]. 

Almost one-third of the average human’s life is spent sleeping [22]. Sleep disorders not only affect the QoL [12,13,23], but also increase the number of road traffic accidents, occupational accidents, risk of errors, and injuries to others as a result of reduced consciousness [22], and a delay in sleep onset of more than 30 minutes almost doubles the risk of mortality after controlling for background variables [23]. Despite this reported direct association between sleep quality and QoL, a cause-and-effect relationship has not yet been established [24].

Nocturnal asthma and sleep apnea are common in asthmatic patients [4]. Severe sleep-related physiological changes in the respiratory system increase the incidence of asthma attacks at night [12]. Studies have indicated poor control of asthma in more than half of asthmatic patients [11]. This may be largely due to the lack of attention to sleep quality and nocturnal symptoms of asthma in the classification, diagnosis, and treatment of patients [18].

Sleep disturbances, especially insomnia, have been proven to be a baseline risk factor for the onset of asthma and its related adverse impacts on health-related QoL [12,15]. On the other hand, asthmatic adults are at a higher risk of developing sleep problems [25]. However, data on the relationship between clinical characteristics and patient perception of health and control of symptoms are not convincing, and some findings are controversial or not tenable in terms of routine practice [11]. Hence, patients would greatly benefit from a study of the main predictors of patient satisfaction with the treatment [9,12,13,18].

The relationship between QoL and sleep quality in patients with asthma has not been seriously investigated in our country. In light of this, this study was conducted to explore this relationship to devise methods to improve the diagnosis, classification, and management of asthmatic patients in Semnan, Iran.

## Materials and methods

This cross-sectional study used convenience sampling to select 76 known cases of asthma who presented for follow-up visits to our university clinic. The inclusion criteria were as follows: patients aged more than 18 years who were diagnosed with asthma by specialists based on spirometry results, forced expiratory volume (FEV1) of less than 80% of the predicted value, FEV1/forced vital capacity (FVC) of less than 0.7, FEV1 change of more than 12% from the initial value, and absolute change in FEV1 of less than 200 mL after taking two to three puffs of a short-acting bronchodilator, The exclusion criteria were the presence of malignancies or other severe incurable diseases such as major depression, congestive heart failure, kidney failure, and liver failure, or insomnia, sleep disorders, or being engaged in shift work. This study was approved by the Ethics Committee of the Semnan University of Medical Sciences, Semnan, Iran (REC.538950).

The patients’ demographic information including age, marital status, education level, and socioeconomic status was collected and recorded after obtaining informed consent. The data collection tools used to assess this relationship were the SF-36 [17] and the Pittsburgh Sleep Quality Index (PSQI) [26]. These tools were developed by experts to investigate QoL and sleep quality. Both questionnaires were completed through interviews conducted by medical doctors.

The SF-36 questionnaire is an excellent and simple tool for investigating QoL in people aged over 14 years [17] and has been used in the study of many chronic diseases [9,20,21,27]. It was first used for asthma by Bousquet et al. in 1993 [17]. It was validated and translated into Persian by Dr. Montazeri in 2005 and introduced as an appropriate tool for the study of QoL [19]. The SF-36 consists of physical and mental component scores (PCS-MCS) with eight subscales including physical function (10 items), bodily pain (two items), social function (two items), mental health (five items), general health (five items), vitality (four items), physical problems (four items), and emotional problems (three items). The scale ranges between scores of 0-100, where 0 means disability and 100 stands for no disability [19].

The nine-item PSQI assesses the patient’s perception of sleep quality over the past month and comprises seven components: subjective sleep quality, sleep latency, sleep duration, sleep efficiency, sleep disturbances, use of sleeping medication, and daytime dysfunction (i.e., daytime problems the patient experiences as a result of insomnia). The items can be rated from 0 (normal), 1 (mild problems), 2 (moderate problems), to 3 (severe problems). The total score is the sum of the scores of the seven scales, ranging from 0 to 21. A PSQI score >5 has been determined to represent poor sleep quality [4]. The validity of this data collection tool has previously been confirmed in the Iranian population [28].

Continuous variables are displayed as means and standard deviation (SD), and tables of frequency and relative frequency are used to describe categorical variables. Analysis of variance (ANOVA), independent t-test, and Pearson’s correlation coefficient were used to analyze the data. All analyses were performed using the SPSS Statistics software version 16 (IBM, Armonk, NY). A p-value <0.05 was considered statistically significant.

## Results

The mean age of the study subjects was 46.8 ± 13.7 years, and the modified sleep quality score was 8.2 ± 3.8. A total of 55 (72.4%) of the subjects had poor sleep quality as per the modified sleep quality score. In this study, a modified PSQI score was used instead of the total PSQI, which was calculated based on all components except for sleep duration as it was the only variable that was not significantly correlated with QoL.

Table 1 shows the demographic characteristics of the study group. PCS and MCS subdimensions of QoL and the modified PSQI score significantly correlated only with age as illustrated.

**Table 1:** General characteristics of asthmatic patients *Significant correlation of higher age with lower MCS and PCS and poor PSQI NS: not significant; MCS: mental component score; PCS: physical component score; PSQI: Pittsburgh Sleep Quality Index

Variable		N (%)	P-value
Gender	Female	43 (56.6)	NS
	Male	33 (43.4)	
Marital status	Single	9 (11.8)	NS
	Married	54 (71.1)	
	Widowed/divorced	13 (16.1)	
Educational level	Illiterate or primary school	11 (14.5)	NS
	High school diploma	39 (51.3)	
	University education	26 (34.2)	
Place of residence	Urban	61 (80.3)	NS
	Rural	15 (19.7)	
Age (years)	Less than 45	36 (47.4)	<0.05*
	45-59	21 (27.6)	
	More than 60	19 (25.0)	

The relationships of the physical and mental dimension scores, as well that of PSQI score, with sex, place of residence, and education level were found to be statistically insignificant. However, ANOVA showed that MCS and PSQI scores were lower in the widowed subjects compared to single and married subjects (p<0.05). The mean ± SD of the mental and physical components of the QoL questionnaire was 51.6 ± 15.9 and 59.0 ± 15.5 respectively. The maximum score for a QoL dimension was observed for bodily pain (73.8 ± 22), and the minimum score was seen for role limitation-emotional (46.0 ± 31).

The mean ± SD of PCS-MCS and all of their subcategories were significantly higher in good sleepers. Table 2 shows correlations across all dimensions as well. The most and the least score gap between subjects with good and poor sleep quality were in emotional role limitation and social function respectively.

**Table 2:** Components of quality of life in people with good and poor sleep quality and the relationship between sleep quality and quality of life dimensions MCS: mental component score; PCS: physical component score; PSQI: Pittsburgh Sleep Quality Index; SD: standard deviation

Variable	Good sleep quality (PSQI <5)	Poor sleep quality (PSQI >5)	Total PSQI	
	Mean ± SD	Mean ± SD	Correlation	P-value
Mental dimensions (MCS)	67.0 ± 13.9	45.7 ± 12.3	-0.56	0.000
Physical dimensions (PCS)	73.6 ± 13.0	53.4 ± 12.4	-0.54	
Physical function	76.8 ± 21.8	46.8 ± 22.5	-0.37	
Bodily pain	92.0 ± 15.7	66.8 ± 20.2	-0.50	
Role limitation-physical	78.6 ± 22.7	53.6 ± 27.8	-0.45	
Role limitation-emotional	79.4 ± 32.4	33.3 ± 35.1	-0.53	
Vitality	69.7 ± 16.7	50.1 ± 17.7	-0.37	
Mental health	67.8 ± 17.9	52.9 ± 15.6	-0.28	0.001
Social function	50.9 ± 9.5	47.2 ± 11.1	-0.16	0.000
General health	47.5 ± 12.8	46.5 ± 8.2	-	0.006

Independent t-test revealed the relationship of all components except social functioning and general health with the sleep quality to be significant. A weak to moderate correlation was found with these variables. The logistic regression analysis showed that PSQI <5 predicted MCS of 64 or more in QoL, as presented in Table 3. This relationship persisted even after adjusting for age.

**Table 3:** Binary logistic regression between PSQI (poor-good) and mental component score (MCS) – SF-36 ^a^Variable(s) entered on step 1: MCS PSQI: Pittsburgh Sleep Quality Index; SF-36: 36-Item Short-Form Health Survey QoL questionnaire

		B	S.E.	Wald	df	Sig.	Exp (B)	95% CI for EXP (B)	
								Lower	Upper
Step 1^a^	MCS	0.113	0.027	17.579	1	0	1.12	1.062	1.18
	Constant	-7.245	1.584	20.932	1	0	0.001		

## Discussion

This study analyzed the efficacy of QoL and sleep quality as indicators in the approach toward and treatment plans of asthma as a chronic disease with a noticeable burden. Our research results indicate that the mean scores of all QoL components were lower in patients with poor sleep quality. Incorporating assessment tools for these two items will improve patients' satisfaction and perception of control.

SF-36 is one of the most reliable tools generally used to study QoL in the general population and chronic diseases [9,17,20,21,27]. Poor QoL (lower PCS-MCS) has been reported in patients with respiratory diseases, especially current asthma [3,4,6,8,18], asthma with allergic comorbidities [3], and sleep disorders [13]. Despite several clinical trials and significant advances in asthma research, treatment plans, and drug production in recent years, patient-related outcomes, especially in terms of real-life aspects such as QoL and sleep quality, have not been incorporated in most of them [3,7]. The relationship between QoL and chronic diseases such as asthma involves a wide range of issues. Neglecting these issues causes poor control of the disease despite treatment advancements [9,15]. Although we used only a non-specific QoL assessment tool, since multimorbidity is unfortunately common in communities, it could improve overall patient satisfaction in general practice.

Among the subscales of QoL, the most preserved score in our study was in bodily pain (PCS), as expected, compared to social function (MCS) in myocardial infarction and heart failure in ischemic heart disease due to the prominence of chest pain [29]. In this study, the lowest subscale was for role emotional (MCS), which is in accordance with the findings of Montazeri et al. in elderly subjects [19] but contrasting with role physical (PCS) in heart failure [29]. Poor diabetes control was correlated with lower physical functioning, and overall PCS below 50 [20]. Another study has shown significantly lower mental health and role limitation-emotional scores in subjects with insomnia even after controlling for psychological diseases [12].

Similar to previous studies, this study found the mean QoL score to be independent of factors such as gender, place of residence, and educational level [6,15]. A study by Cappa et al. also identified QoL in patients as a determining factor for the appropriate classification and control of diseases such as asthma [6]. Another study has reported a negative relationship of age with the mean physical and mental QoL scores [12]. The importance of demographic variables cannot be denied in epidemiologic studies, but the use of QoL and sleep in assessment bring up more modifiable risk factors and make more sense in “personalized medicine”. Poor sleep quality was also reported to be a strong independent predictor of poor QoL in healthy people, patients with systemic lupus erythematosus [21], ischemic heart disease [27], and patients with asthma [7,12,18] and chronic obstructive pulmonary disease (COPD) [16,24].

The study of a gap between treatment protocols and desirable control of asthma highlighted the importance of the effects of nocturnal asthma and sleep disorders on QoL in this group of patients [7]. Sleep quality has been shown to be an important predictor of daily function and QoL in asthmatic patients. The prevalence and mean score of sleep quality were found to be higher and more adverse in our patients than in multiple sclerosis patients (72% vs. 70%; 8.2 vs. 5.2) [30]. Poor sleep quality could forecast a lower QoL score in ischemic heart disease [27] and diabetes type 2 [20], but not in cardiac surgery [27]. Asthma leads to more night-time symptoms and consequently lower QoL even in the absence of overt pain and disability.

Asthma-specific studies estimate the prevalence of sleep disorders to be from 51% [18] to more than 90% [12]. Braido et al. found that poor control of asthma is caused by excessive nocturnal airway disturbances and apnea in asthmatic patients [4]. Investigation of sleep quality and recording and controlling the nocturnal symptoms of the disease, therefore, play a key role in the treatment of asthma. The emergence of nocturnal symptoms was found not to be correlated with the severity of asthma. In a 12-year follow-up study, sleep disorders were identified as one of the important factors in the categorization and control of asthma [11]. Both nocturnal symptoms and poor sleep quality were found to be correlated with poor control of the patient’s asthma and poor QoL [15]. This indicates that the relationship between QoL and sleep quality in asthmatic patients is independent of the treatment program's specificity and accuracy, somewhat confirming the results of our study, which indicated that this relationship is independent of the duration of the disease, based on SF-36 and PSQI.

Although the latest standard diagnosis methods and drug treatment protocols were used, the physicians were not involved in dealing with the real-life aspects: sleep quality, personalized medicine. On the other hand, patients think that such disturbances/symptoms are an integral part of the disease and there is no way to overcome them and that they should suffer!

This study has several limitations. Firstly, the asthma QoL questionnaire (AQLQ) has not yet been validated and translated into Persian. Also, this study was performed among outpatient asthmatics based on their documents and patient-reported information, and we had limited time for interviews. Additionally, the generalization of the results is limited due to the cross-sectional design of the study.

## Conclusions

Poor sleep quality was common in our patients and was associated with a lower overall and disease-specific QoL index. It is recommended that sleep quality and QoL assessment should be included in the medical approach plan and used individually to increase patient satisfaction. Considering health-related QoL as an index of successful management of asthma should take sleep quality into account to increase patient satisfaction and orient the approach toward personalized medicine. Appraisal of these indexes also promotes a better understanding of the disease burden.

## Figures and Tables

**Figure 1:**
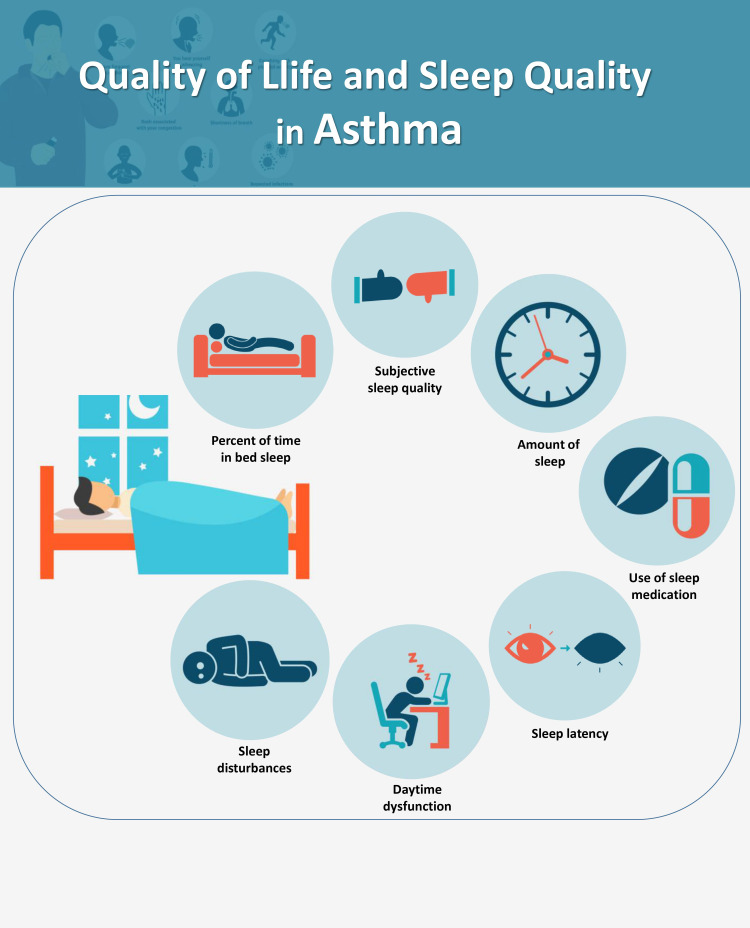
Graphical abstract The graphical abstract was obtained from an independent designer and he has stated that it is not a copy

## References

[1] (2022). Global Initiative for Asthma. Global strategy for asthma management and prevention, 2019. http://10.1183/13993003.00598-2019.

[2] GBD 2015 Chronic Respiratory Disease Collaborators (2017). Global, regional, and national deaths, prevalence, disability-adjusted life years, and years lived with disability for chronic obstructive pulmonary disease and asthma, 1990-2015: a systematic analysis for the Global Burden of Disease Study 2015. Lancet Respir Med.

[3] Lee LK, Obi E, Paknis B, Kavati A, Chipps B (2018). Asthma control and disease burden in patients with asthma and allergic comorbidities. J Asthma.

[4] Braido F, Baiardini I, Ghiglione V, Fassio O, Bordo A, Cauglia S, Canonica GW (2009). Sleep disturbances and asthma control: a real life study. Asian Pac J Allergy Immunol.

[5] Varmaghani M, Farzadfar F, Sharifi F (2016). Prevalence of asthma, COPD, and chronic bronchitis in Iran: a systematic review and meta-analysis. Iran J Allergy Asthma Immunol.

[6] Cappa V, Marcon A, Di Gennaro G (2019). Health-related quality of life varies in different respiratory disorders: a multi-case control population based study. BMC Pulm Med.

[7] Braido F, Baiardini I, Ferrando M (2021). The prevalence of sleep impairments and predictors of sleep quality among patients with asthma. J Asthma.

[8] Juniper EF, Norman GR, Cox FM, Roberts JN (2001). Comparison of the standard gamble, rating scale, AQLQ and SF-36 for measuring quality of life in asthma. Eur Respir J.

[9] Böhmer MM, Brandl M, Brandstetter S, Finger T, Fischer W, Pfeifer M, Apfelbacher C (2017). Factors associated with generic health-related quality of life in adult asthma patients in Germany: cross-sectional study. J Asthma.

[10] Megari K (2013). Quality of life in chronic disease patients. Health Psychol Res.

[11] Ilmarinen P, Juboori H, Tuomisto LE, Niemelä O, Sintonen H, Kankaanranta H (2019). Effect of asthma control on general health-related quality of life in patients diagnosed with adult-onset asthma. Sci Rep.

[12] Luyster FS, Teodorescu M, Bleecker E (2012). Sleep quality and asthma control and quality of life in non-severe and severe asthma. Sleep Breath.

[13] Lin YC, Lai CC, Chien CC (2017). Is insomnia a risk factor for new-onset asthma? A population-based study in Taiwan. BMJ Open.

[14] Kia NS, Malek F, Ghods E, Fathi M (2017). Health-related quality of life of patients with asthma: a cross-sectional study in Semnan, Islamic Republic of Iran. East Mediterr Health J.

[15] Jansson SA, Axelsson M, Hedman L, Leander M, Stridsman C, Rönmark E (2016). Subjects with well-controlled asthma have similar health-related quality of life as subjects without asthma. Respir Med.

[16] Shah A, Ayas N, Tan WC (2020). Sleep quality and nocturnal symptoms in a community-based COPD cohort. COPD.

[17] Bousquet J, Knani J, Dhivert H, Richard A, Chicoye A, Ware JE Jr, Michel FB (1994). Quality of life in asthma. I. Internal consistency and validity of the SF-36 questionnaire. Am J Respir Crit Care Med.

[18] Panigrahi MK, Padhan M, Mohapatra PR (2019). Sleep quality, asthma control and quality of life in adults with asthma - a cross-sectional study. Eur Respir J.

[19] Montazeri A, Goshtasebi A, Vahdaninia M, Gandek B (2005). The Short Form Health Survey (SF-36): translation and validation study of the Iranian version. Qual Life Res.

[20] Bani-Issa W, Al-Shujairi AM, Patrick L (2018). Association between quality of sleep and health-related quality of life in persons with diabetes mellitus type 2. J Clin Nurs.

[21] Kotterba S, Neusser T, Norenberg C, Bussfeld P, Glaser T, Dörner M, Schürks M (2018). Sleep quality, daytime sleepiness, fatigue, and quality of life in patients with multiple sclerosis treated with interferon beta-1b: results from a prospective observational cohort study. BMC Neurol.

[22] (2006). Sleep Disorders and Sleep Deprivation: An Unmet Public Health Problem. https://books.google.co.in/books/about/Sleep_Disorders_and_Sleep_Deprivation.html?id=KexmhhRu84MC&source=kp_book_description&redir_esc=y.

[23] Dew MA, Hoch CC, Buysse DJ (2003). Healthy older adults' sleep predicts all-cause mortality at 4 to 19 years of follow-up. Psychosom Med.

[24] Vukoja M, Kopitovic I, Milicic D, Maksimovic O, Pavlovic-Popovic Z, Ilic M (2018). Sleep quality and daytime sleepiness in patients with COPD and asthma. Clin Respir J.

[25] Sundbom F, Lindberg E, Bjerg A (2013). Asthma symptoms and nasal congestion as independent risk factors for insomnia in a general population: results from the GA(2)LEN survey. Allergy.

[26] Buysse DJ, Reynolds CF 3rd, Monk TH, Berman SR, Kupfer DJ (1989). The Pittsburgh Sleep Quality Index: a new instrument for psychiatric practice and research. Psychiatry Res.

[27] Wang S, Xin HN, Chung Lim Vico C, Liao JH, Li SL, Xie NM, Hu RF (2020). Effect of an ICU diary on psychiatric disorders, quality of life, and sleep quality among adult cardiac surgical ICU survivors: a randomized controlled trial. Crit Care.

[28] Farrahi Moghaddam J, Nakhaee N, Sheibani V, Garrusi B, Amirkafi A (2012). Reliability and validity of the Persian version of the Pittsburgh Sleep Quality Index (PSQI-P). Sleep Breath.

[29] Huber A, Oldridge N, Höfer S (2016). International SF-36 reference values in patients with ischemic heart disease. Qual Life Res.

[30] Tabrizi FM, Radfar M (2015). Fatigue, sleep quality, and disability in relation to quality of life in multiple sclerosis. Int J MS Care.

